# The 2023 WCIRDC: Obesity

**DOI:** 10.1111/1753-0407.13568

**Published:** 2024-04-23

**Authors:** Zachary T. Bloomgarden

**Affiliations:** ^1^ Department of Medicine, Division of Endocrinology Diabetes and Bone Disease Icahn School of Medicine at Mount Sinai New York New York USA

The 21st annual World Congress on Insulin Resistance, Diabetes and Cardiovascular Disease, held in Los Angeles, California on December 7–9, 2023, included 69 presentations spanning a myriad of aspects of diabetes and its complications, atherosclerosis, renal disease, liver disease, and novel therapeutic approaches. This second summary focuses on presentations at the meeting pertaining to obesity.

Philipp Scherer (Dallas, Texas) noted that, similarly to the importance of fibrosis in metabolic dysfunction‐associated fatty liver disease (MAFLD), which may persist after steatosis has been treated and which underlies the development of cirrhosis, obesity is associated with increased localized fibrosis and disrupted angiogenesis in adipose tissue, mediated by low levels of adiponectin and increased production of leptin, steroid hormones, and inflammatory mediators.^1^ Scherer highlighted the role of endotrophin, a cleavage product of collagen that may mediate fibrosis in the liver, kidneys, and heart. Development of agents to neutralize this peptide might have therapeutic benefits. He also showed studies suggesting that the greater clinical potency of tirzepatide than of the glucagon‐like peptide (GLP)‐1 receptor agonist (RA) semaglutide may be an effect of glucose‐dependent insulinotropic polypeptide (GIP) receptor activation in increasing energy expenditure.

Richard Bergman (Los Angeles, California) discussed the use of the body mass index (BMI) in quantitating obesity, explaining that the measure derives from the work of Adolphe Quetelet, who developed the concept of the “Average Man” in the nineteenth century. He proposed use of an index based on the observation that weight varied in proportion to the square of height. During the twentieth century the term BMI was popularized by Ancel Keys, based on studies showing that the Quetelet index correlated with direct measurements of body fat. The BMI does not, however, give information about fat distribution, and Bergman suggested that it is not a good measure of body fat, giving no information about the mechanisms operative in a given individual. Measurement of skinfold thicknesses, the use of BMI in conjunction with waist circumference, underwater weighing, and the more recent body adiposity index (calculated as hip/height^1.48) have been proposed. Bergman reviewed his work in population studies with dual‐energy X‐ray absorptiometry measurement of fat mass. Analysis of a variety of possible relationships between sex, height, weight, and waist circumference led Bergman to propose a new measure, relative fat mass (RFM), calculated as: RFM = 64 – (20*Height/WC) + (12*sex), with sex = 0 in men and sex = 1 (women).^2^, ^3^ Bergman reviewed studies showing good prediction of risks of diabetes, heart failure, and coronary disease with this measure.

Samuel Klein (St. Louis, Missouri) discussed the complex relationships between BMI and cardiovascular disease, pointing out the concepts of metabolically healthy vs unhealthy normal weight, overweight, and obesity, with a metabolically unhealthy person having greater improvement than the metabolically healthy one from a given degree of weight loss,^4^ so that obesity may not itself be the mediator of adverse outcomes. Among people with relatively early type 2 diabetes (T2D), diet can effectively lead to remission,^5^ with progressively greater degrees of weight loss leading to progressively greater improvement in insulin sensitivity,^6^ leading Klein to argue that the “primary first step should be aggressive weight loss management.” Klein reviewed the Swedish Obesity Study, which showed lower mortality, and fewer cardiovascular (CV) and malignancy outcomes beginning 6 years after bariatric surgery than those seen in persons not electing to have such surgery,^7^ and the more recent study showing that high‐risk persons with obesity had fewer adverse CV outcomes when treated with semaglutide than with lifestyle intervention alone.^8^ The Look AHEAD trial of persons with T2D showed, however, that despite progressive improvement in CV risk factors with greater weight loss CV outcome benefit was not demonstrated,^9^ so that lifestyle approaches to weight loss management are probably insufficient for the majority of persons with obesity.

Eric Ravussin (Baton Rouge, Louisiana) discussed calorie restriction with intermittent fasting, reviewing the use of time‐restricted eating as a strategy to restrict calories, pointing out that in interviews people report eating over a period of 12 hrs per day and that smartphone data actually suggest 15 hrs to be more accurate. The principle underlying intermittent fasting is reversing to 15–16 hrs per day of not eating. A comparison of eating equivalent quantities over 6 rather than 12 h showed improvement in insulin sensitivity, blood pressure, and oxidative stress.^10^ A meta‐analysis of time‐restricted eating showed, however, that there was short‐ but not long‐term weight loss with this approach.^11^ Of note, a poster at the American Heart Association's scientific meeting in March 2024 analyzing dietary patterns among 43 849 participants in the National Health and Nutrition Examination Surveys from 2003 to 2018 showed an increase in CV mortality among those reporting eating duration <8 h/day,^12^ further suggesting the need for caution in advising this approach. Related approaches include alternate day fasting, 1‐day‐per‐week fasting, and alternate day modified fasting. Ravussin discussed a “new thing on the horizon,” precision nutrition based on predicting a given individual's responses to different foods and to different dietary patterns based on genomic and food intake analysis.

Ania Jastreboff (New Haven, Connecticut) and Richard Pratley (Orlando, Florida) reviewed developments in use of nutrient‐stimulated hormone‐based therapies for obesity, with potential agents being developed based on peptide YY, oxyntomodulin, amylin, and glucagon as well as GIP and GLP‐1 (Table 1). All show promising weight loss effects, with the goal being to move beyond weight reduction to optimizing health outcomes. Animal models have shown a variety of potential actions of these agents on inflammation and on the kidney, lungs, brain, liver, and adipocytes. A number of agents are under study,^13^ including the amylin receptor agonist, cagrilintide, combined with the GLP‐1RA semaglutide,^14^ survodutide, a glucagon/GLP‐1 receptor dual agonist,^15^ and retatrutide,^16^ a single peptide with agonist activity at the GIP, GLP‐1, and glucagon receptors. Oral agents being developed include orforglipron, a nonpeptide GLP‐1 receptor agonist,^17^ and high‐dose oral semaglutide.^18^ Maridebart cafraglutide is being developed as a monthly injection, acting as a GLP‐1 RA and an antagonist of the GIP receptor.^19^ A hypothesis being advanced to explain potential benefit of GIP receptor antagonism is that chronic GIP receptor agonism actually downregulates GIP response, acting as an antagonist.^20^

**TABLE 1:** Agents being developed for weight loss.

Agent	Brand name	Administration	Manufacturer	% Weight loss^a^	GLP‐1 receptor action	GIP receptor action	Other receptor action
Semaglutide	Wegovy	Weekly, parenteral	Novo Nordisk	12.4	Activation	None	None
Semaglutide		Oral	Novo Nordisk	15.6			
Tirzepatide	Zepbound	Weekly, parenteral	Eli Lilly	20.1	+	+	None
Liraglutide	Saxenda	Daily, parenteral	Novo Nordisk	4	+	None	None
Maridebart cafraglutide	MariTide	Monthly, parenteral	Amgen		+	Inhibit	None
Danuglipron		Daily, oral	Pfizer		+	None	None
Orforglipron		Daily, oral	Eli Lilly	12.4	+	None	None
VK2735		Weekly, parenteral	Viking Therapeutics		+	+	None
VK2375		Daily, oral	Viking Therapeutics		+	+	None
Pemvidutide		Altimmune	Weekly, parenteral	13.4	+	+	None
GSBR‐1290		Weekly, oral	Structure Therapeutics		+	None	None
Survodutide		Zealand Pharma, Boehringer Ingelheim	Weekly, parenteral	16.7	+	None	Glucagon +
Retatrutide		Weekly, parenteral	Eli Lilly	22.1	+	+	Glucagon +
Cagrilintide + semaglutide	CagriSema	Weekly, parenteral	Novo Nordisk	17.1^b^	+	None	Amylin +
Efino‐pegdutide		Weekly, parenteral	Merck	10.0	+	None	Glucagon +
Mazdutide		Weekly, parenteral	Innovent	15.4^b^	+	None	Glucagon +

Abbreviations: GIP, glucose‐dependent insulinotropic polypeptide; GLP‐1, glucagon‐like peptide 1.

^a^ Weight loss in persons not having diabetes.

^b^ Not placebo corrected.

Obesity increases liver fat accumulation, with Rohit Loomba (La Jolla, California) noting that steatotic liver disease (SLD) is the new “umbrella term,” including metabolic dysfunction‐associated SLD (MASLD), metabolic dysfunction associated steatohepatitis (MASH), alcohol‐associated liver disease (ALD), and MASLD associated with increased alcohol intake (MetALD). Globally, MASLD affects 25% of the adult population and 65% of persons with T2D, with 14% of those with T2D having advanced fibrosis and 6% having cirrhosis. Loomba reviewed his study of risk factors for advanced fibrosis among relatives of persons with MASLD: age, male sex, diabetes, and advanced fibrosis in a relative.^21^ Loomba reviewed treatment approaches, lifestyle approaches including avoidance of excess heavy alcohol use, persons with fibrosis completely eliminating alcohol, with the interesting observation that drinking at least two cups of coffee daily may be beneficial. A variety of pharmacologic approaches have been studied, including pioglitazone., vitamin E, and statins. The farnesoid X‐activated receptor agonist obetocholic acid is used in treatment of primary biliary cholangitis but was found not to significantly improve MAFLD, with the important side effects of increasing low‐density lipoprotein cholesterol and causing cholestatis, leading to pruritus and increased gallstone formation. Semaglutide 2.4 mg weekly improves MASH but does not reverse cirrhosis, although studies in stage 2 and Stage 3 fibrosis are in progress. The fibroblast growth factor 21 analog pegozafermin significantly improved MASH and fibrosis in a phase 2b trial,^22^ with further studies in progress. A final agent is resmetirom, a thyroid hormone receptor‐beta agonist, which has just received conditional Food and Drug Administration approval (pending confirmatory trials) for use in MASH with stages F2 and F3 fibrosis, based on a Stage 3 trial showing improvement in fibrosis.^23^ A recent meta‐analysis suggested efficacy for SLD (but not for fibrosis) of the milk thistle‐derived supplement silymarin.^24^
